# Severity of Acute Drug Poisonings Treated in the Pediatric Emergency Department of a Hospital in Western Mexico

**DOI:** 10.3390/medsci14020326

**Published:** 2026-06-17

**Authors:** Ruth Yesica Ramos-Gutiérrez, Mireya Robledo-Aceves, Santiago José Guevara-Martínez, Nelson Bruno de Almeida-Cunha, Raymundo Escutia-Gutiérrez, Martin Zermeño-Ruiz, Karla Valeria Díaz-Rivera, Ángel Abad Del Río-Chávez, César Ricardo Cortez-Álvarez, Alfredo Fernando Cortez-Martínez, Damian Fierros-Uribe, Héctor Andrés González-Ruiz, Rebeca Escutia-Gutiérrez

**Affiliations:** 1 Division of Pediatrics, “Dr. Juan I. Menchaca” Civil Hospital of Guadalajara, Guadalajara 44340, Jalisco, Mexico; ryramosg@hcg.gob.mx (R.Y.R.-G.); mrobledo@hcg.gob.mx (M.R.-A.); 2 Department of Pharmacobiology, University Center of Exact Sciences and Engineering, University of Guadalajara, Guadalajara 44430, Jalisco, Mexico; santiago.guevara@academicos.udg.mx (S.J.G.-M.); bruno.dealmeida@academicos.udg.mx (N.B.d.A.-C.); raymundo.escutia@academicos.udg.mx (R.E.-G.); martin.zermeno@academicos.udg.mx (M.Z.-R.); cesar.cortez@academicos.udg.mx (C.R.C.-Á.); alfredo.cortez7546@alumnos.udg.mx (A.F.C.-M.); damian.fierros@academicos.udg.mx (D.F.-U.); 3 La Cienega University Center, University of Guadalajara, Ocotlan 47810, Jalisco, Mexico; karla.diaz3193@alumnos.udg.mx; 4 Bromatology Laboratory, Faculty of Agrobiology “Presidente Juárez”, Michoacan University of San Nicolas de Hidalgo, Uruapan 60170, Michoacán, Mexico; abad.del.rio@umich.mx; 5 Research Coordination Unit, Jalisco Institute for Pain Relief and Palliative Care, Jalisco Ministry of Health, Zapopan 45150, Jalisco, Mexico; palia.investigacion@ssj.gob.mx; 6 Department of Molecular Biology and Genomics, University Center of Health Sciences, University of Guadalajara, Guadalajara 44340, Jalisco, Mexico

**Keywords:** drug poisoning, pediatrics, emergency care

## Abstract

Background: Acute drug poisoning is one of the leading causes of admission to pediatric emergency departments and represents a significant public health concern because of its potential severity and associated morbidity and mortality. This study aimed to describe the clinical, epidemiological, and severity characteristics of pediatric patients with acute drug poisoning treated at a tertiary care hospital in western Mexico. Methods: A retrospective, observational, descriptive study was conducted in the pediatric emergency department of Nuevo Hospital Civil de Guadalajara “Dr. Juan I. Menchaca” from January 2016 to December 2024. Results: The mean age of the patients was 77.1 months, with a predominance of females (61.9%). Most poisoning events (97.1%) occurred in the home. Accidental poisoning was the most frequent mechanism (54.5%), followed by suicide attempts (24.4%) and drug overdoses (17.6%). Regarding medical care, 50% of patients arrived at the emergency department within the first four hours after exposure, and 55.1% had a hospital stay of less than 12 h. The most involved drug groups were anxiolytics, mainly benzodiazepines (21.6%), followed by polypharmacy (17.6%) and antiemetic use (13.6%). The most frequent toxidrome was hypnotic–sedative syndrome (42.6% of cases). Multivariate analysis showed that exposure to anticonvulsants was significantly associated with a longer hospital stay (odds ratio [OR] = 7.31, *p* = 0.003). Most cases were classified as mild according to the Poisoning Severity Score, and no deaths were reported. Conclusions: Although pediatric drug poisoning generally has a favorable prognosis, it remains a significant public health issue. These findings highlight the need for targeted preventive strategies, including caregiver education, safe medication storage at home, and increased awareness and training programs for both families and healthcare professionals.

## 1. Introduction

Acute drug poisoning is an important cause of morbidity in the pediatric population [1]. Several studies have reported that this type of poisoning is among the leading causes of admission to emergency departments [2,3,4]. Recent reports from the American Association of Poison Control Centers (AAPCC) confirm that pharmaceutical exposure continues to represent one of the most frequent categories of toxic exposure in children and adolescents, accounting for a substantial proportion of emergency consultations [5,6].

Acute poisoning is characterized by the rapid absorption of a chemical agent following a single or multiple doses, resulting in the development of a clinical condition over a short period (≤24 h). Its effects appear quickly, and the outcome is typically full recovery or death. In pediatric populations, acute drug poisoning often presents with rapidly developing clinical manifestations, frequently expressed through toxidromes, such as anticholinergic, serotonergic, or sedative–hypnotic syndromes, depending on the substance involved [7]. Clinical evolution varies according to the agent, ingested dose, and timeliness of medical care, ranging from complete recovery with prompt treatment to potentially life-threatening complications [1,8]. Standardized tools, such as the Poisoning Severity Score (PSS), have been increasingly used to classify clinical severity and improve comparability among studies [9]. In medication poisoning, the oral route is the most frequent, accounting for approximately 98% of cases [10], consistent with large surveillance databases [11].

Throughout history, poisonings have posed a persistent threat to human health and have been a constant challenge for healthcare systems in terms of prevention [12]. Accidental poisoning is most common among children under five years of age. The identified risk factors for this age group include curiosity about their environment and a tendency to imitate, explore, and emulate adult behavior [13]. Unsupervised medication exposure in the home environment remains a leading cause of emergency department visits among this age group [14]. Conversely, intentional poisoning is more frequently observed in adolescents [13,15], occurring as self-harm or recreational intent. Recent international data indicate a concerning rise in intentional self-poisoning and drug overdoses among adolescents and young adults [16]. In some cases, poisoning may be related to child maltreatment [17]. Established preventive strategies in the pediatric population include safe storage practices, parental education regarding appropriate medication handling, and the use of child-resistant packaging, which has been effective in reducing severe exposures [18].

Drug poisoning in pediatric patients can be severe and potentially life-threatening; therefore, it is essential for pediatricians to be familiar with the clinical effects of frequently implicated medications. Effective management also requires knowledge of highly toxic medications, the ingestion of which, even in minimal quantities, may be lethal owing to their intrinsic toxicity or high commercial concentration [19]. Large-scale surveillance studies continue to highlight the importance of early recognition and risk stratification of pediatric pharmaceutical exposure [5,20].

The epidemiology of pediatric poisonings varies across studies, with reported prevalence ranging from 0.33% to 7.6%. According to Bezai Morales et al. [10], medication poisoning accounted for 0.08% of cases in their study. Several studies have explored the epidemiological and clinical profiles of pediatric intoxication. Several studies have explored the epidemiological and clinical profiles of pediatric intoxications in different regions, including Latin America, where medication exposure remains one of the most common causes [21,22]. For instance, a retrospective analysis conducted between 2016 and 2020 in a hospital in western Mexico documented 459 cases in patients under 16 years of age, with an overall prevalence of 3.16%, of which 27.6% involved medication ingestion. This study, led by Robledo Aceves et al. [23], also found that children under five years of age were mainly poisoned by medications, hydrocarbons, and household products (*p* = 0.03), whereas older children are more commonly presented with incidents involving envenomation by venomous animals or exposure to psychoactive substances. Additionally, research on diagnostic errors in the pediatric emergency department at Hospital Juárez in Mexico City (2007–2012) highlighted the challenges of early recognition and underreporting of intoxication cases. Although this study did not employ the Poisoning Severity Score (PSS), it provided relevant context regarding structural barriers to clinical care. At our institution, poisonings are reported with a prevalence of 3% [23], placing them among the top ten causes of emergency department admissions, consistent with previous reports [12,15].

In Mexico, there has been a documented increase in the prescription of psychotropic drugs to treat anxiety and sleep disorders in adults. This trend has significantly increased the availability of high-risk medications in the domestic environment. Given that safe storage practices are often inconsistent, benzodiazepines have become one of the most accessible agents for both accidental ingestion in young children and self-harm in adolescents [24].

Despite the recognized burden of pediatric drug poisoning, significant knowledge gaps persist in Mexico, particularly regarding recent severity-focused epidemiological data from its western regions. Most national reports have primarily described general poisoning patterns without systematically characterizing the potential sources of poisoning or assessing the clinical severity of poisoning using standardized instruments, such as the Pittsburgh Poison Center Prescriber Service (PPS). This limitation impedes the capacity to execute precise risk stratification and formulate targeted prevention and management strategies in pediatric emergency settings. Therefore, the present study aimed to describe the clinical severity and epidemiological characteristics of acute drug poisoning cases treated in the pediatric emergency department of a referral hospital in western Mexico, providing updated regional evidence to support clinical decision-making and public health interventions.

## 2. Materials and Methods

### 2.1. Study Design and Setting

A retrospective, observational, descriptive study was conducted to analyze cases of hospitalization through the pediatric emergency department between January 2016 and December 2024. The research was conducted at the “Dr. Juan I. Menchaca” Civil Hospital of Guadalajara, a public institution that provides secondary and tertiary care. This hospital manages more than 15,000 emergency visits annually and serves as a referral center for poisoning cases in Western Mexico.

### 2.2. Participants

The study population comprised girls, boys, and adolescents aged 30 days to 16 years residing in the Guadalajara Metropolitan Area with a confirmed diagnosis of acute drug poisoning. Eligible patients were consecutively identified from the hospitalization records of the pediatric emergency department of our institution. Patients were included if they met the age criteria, lived in the metropolitan area, had a confirmed diagnosis of acute drug poisoning, and were admitted during the study period. The exclusion criteria encompassed patients with toxic exposures of a non-medication etiology, such as ingestion of household cleaning products, pesticides, carbon monoxide poisoning, and envenomation by venomous animals (e.g., snakebites or scorpion stings), as these cases fell outside the scope of acute drug poisoning evaluated in the present study; when suspected drug poisoning was ruled out during hospitalization; when medical records were incomplete; when poisoning due to illicit drugs was confirmed by toxicology screening; or when exposure to medications could not be established. The decision to admit patients was made by pediatricians or pediatric emergency physicians according to the institutional clinical criteria.

### 2.3. Data Sources and Variables

Clinical records were systematically reviewed using a standardized data collection approach. The variables obtained included demographic data, such as sex and age, and sociodemographic information on parents and primary caregivers. The clinical and exposure-related variables included the date of admission, chief complaint, toxic substance involved, mechanism of poisoning (categorized as accidental, intentional, recreational, suicide attempt, or overdose), route of exposure, location of the event, use of folk remedies to alleviate poisoning symptoms, time elapsed between exposure and arrival at the emergency department, and admission shift categorized as morning (08:00–14:00), afternoon (14:00–21:00), night (21:00–08:00), or extended weekend/holiday shift. Information regarding the presence of toxidromes, complementary diagnostic tests, treatment provided in the emergency department (including initial stabilization, gastrointestinal decontamination, and antidote use), patient disposition, and clinical outcomes (survival or death) was also collected. Based on the admission date, the cases were classified by season: spring (March–May), summer (June–August), autumn (September–November), and winter (December–February).

### 2.4. Operational Definitions

Acute poisoning was defined as a pathological process characterized by specific clinical signs and symptoms caused by exposure to a toxic chemical substance capable of producing morphological, functional, or biochemical alterations, with exposure occurring within 24 h of presentation. Age groups were categorized according to the Mexican Official Standard NOM-008-SSA2-1993 as young infants (30 days to 12 months), older infants (1 year to 1 year and 11 months), preschoolers (2–4 years), school-aged children (5–9 years), and adolescents (10–19 years). However, the maximum age included in the analysis was 15 years and 11 months, which corresponds to the institutional upper age limit for admission to the pediatric emergency department. Time from exposure to medical care was categorized into the following intervals: less than 1 h, 1–4 h, 4–8 h, 8–12 h, 12–24 h, and more than 24 h. Length of stay in the emergency department was classified as less than 12 h, 12–24 h, or greater than 24 h.

### 2.5. Treatment and Diagnosis of Poisoning

The management of poisoning was determined by the substance ingested, estimated dose, and clinical presentation. The diagnosis was confirmed based on parental or caregiver reports, physical identification of the medication container brought to the emergency department, and the presence of specific toxidromes. While toxicological screening is the gold standard, in our clinical setting, diagnosis relied on this comprehensive triad of findings. This approach is consistent with emergency management in resource-limited settings, where rapid laboratory confirmation is not always available for all substances. Depending on the amount ingested and the time elapsed since exposure, the treatment protocol included gastric lavage or administration of activated charcoal. Since there is no universal protocol, each case was managed according to the patient’s specific clinical evolution, which influenced the length of hospital stay.

### 2.6. Severity Assessment

Poisoning severity was assessed using the Poisoning Severity Score, a standardized scale that qualitatively evaluates morbidity associated with toxic exposure [24]. It is widely applied in clinical and epidemiological studies to assess poisoning-related morbidity and allow the comparability of data between different centers. The score was determined according to the most severe clinical manifestations observed during the poisoning course, based on objective signs and symptoms affecting different organ systems. According to the original authors, severity was graded into five categories: none (0), minor (1), moderate (2), severe (3), and fatal (4).

### 2.7. Drug Classification

Medications were classified in two ways to determine their association with the type of poisoning. Drugs were grouped by pharmacological category as follows: analgesics, anxiolytics, antipsychotics, antihistamines, anticonvulsants, antiemetics, antihypertensives, anti-inflammatory agents, antitussives and mucolytics, hypoglycemic agents, polypharmacy, and others (including antineoplastics, antacids, cold preparations, anti-infectives, antiparkinsonian drugs, mood stabilizers, hormone replacement therapies, and diuretics).

Second, medications were classified according to their regulatory category based on purchase and dispensing requirements, in accordance with Article 226 of the General Health Law. The resulting groups were as follows: drugs requiring a special prescription (I), drugs requiring a medical prescription that must be retained at the pharmacy with registration in controlled substance logs (II), drugs requiring a medical prescription that may be filled up to three times with controlled substance log registration (III), drugs requiring a medical prescription that may be refilled as indicated by the prescribing physician (IV), over-the-counter drugs authorized for sale exclusively in pharmacies (V), and drugs that do not require a prescription and may be purchased in any commercial establishment (VI).

### 2.8. Bias Control

Selection bias was minimized by including consecutive eligible patients throughout the study period. Information bias was reduced through standardized data extraction procedures from clinical records and by excluding records that lacked key variables necessary for the analysis.

### 2.9. Statistical Analysis

Descriptive statistics were used to summarize the sociodemographic and clinical variables. Absolute (*n*) and relative (%) frequencies were calculated for the categorical variables. Associations between sociodemographic characteristics and poisoning-related parameters were assessed using Pearson’s chi-square or Fisher’s exact test, as appropriate. An ordinal logistic regression model was constructed to evaluate the factors associated with the length of hospital stay in the emergency department (<12, 12–24, and >24 h). The independent variables included age group, sex, and pharmacological classification. Crude and adjusted odds ratios (ORs) with 95% confidence intervals (95% CIs) were estimated. Statistical significance was set at *p* < 0.05. Statistical analyses were performed using IBM SPSS Statistics for Windows version 20.0 (IBM Corp., Armonk, NY, USA).

### 2.10. Ethical Considerations

The study was conducted in accordance with the Declaration of Helsinki and was approved by the Ethics Committee of the “Dr. Juan I. Menchaca” Civil Hospital of Guadalajara (protocol code 33/HCJIM-JAL/24; approved on 14 March 2024). Given its retrospective design and use of anonymized clinical records, the requirement for written informed consent was waived by the Ethics Committee. All procedures complied with the ethical principles outlined in the Declaration of Helsinki and the applicable national regulations governing research involving human subjects. Patient confidentiality was strictly maintained throughout this study.

## 3. Results

During the study period, 204 patients were admitted with poisoning diagnoses. However, 176 patients were included in the final analysis, as 28 patients met at least one of the exclusion criteria and were therefore excluded.

Among the 176 patients included, the mean age was 77.09 months (±62.47), and 109 (61.9%) were female. The Poisoning Severity Score (PSS) indicated that most cases were mild, with a mean score of 0.85 (±0.49) [24]. Nearly half of the patients (40.3%) arrived at the emergency department within 1–4 h of the poisoning event, and 97 patients (55.1%) remained in the emergency department for <12 h.

Regarding the time of admission, 48.2% of cases (n = 85) were admitted during the night shift, followed by the afternoon (37.5%, n = 66), morning (10.2%, n = 18), and extended weekend/holiday shifts (3.9%, n = 7). Regarding the location of exposure, 97.1% of the cases (n = 171) occurred at home, whereas only five events were reported to have occurred in public areas.

### 3.1. Sociodemographic Characteristics

Regarding sociodemographic characteristics, the primary caregivers were both parents in most cases (n = 102). The most frequent level of maternal education was completion of secondary school (n = 62), similar to that of fathers (n = 52). Regarding occupation, most mothers were homemakers (n = 86), whereas most fathers were employed (n = 76). Drug use was reported by eight mothers and 17 fathers in this study. The predominant socioeconomic class was low in 79.5% of the sample (n = 140). In this study, socioeconomic status was established based on national benchmarks from the National Institute of Statistics and Geography (INEGI), drawing on indicators from the National Council for the Evaluation of Social Development Policy (CONEVAL) and internationally accepted public health criteria, which primarily consider income, educational attainment, occupation, housing conditions, and access to basic services. Priority was given to variables with the greatest epidemiological relevance and availability of reliable data, allowing stratification consistent with the scientific literature. Parameters not included were those deemed unfeasible to measure in the study population or lacking solid evidence as determinants of socioeconomic status in health.

Regarding treatment, 22 patients (12.5%) used some type of folk remedy before seeking medical care to alleviate poisoning symptoms, with milk being the most common remedy (63.64%). In 41 cases (23.29%), the toxic agent was identified through laboratory testing, gastric lavage was performed in 47 cases (26.7%), and activated charcoal was administered in 78 patients (44.3%) as a preventive measure to reduce toxin absorption. Only one patient (0.57%) required admission to the intensive care unit, and no deaths were recorded. The use of folk remedies prior to emergency department admission was minimal (12.5%), with milk being the most frequently used remedy (8.2%). Additional sociodemographic and treatment-related variables are presented in Table 1.

In addition, the most common category of toxidromes observed in the patients was hypnotic/sedative (75 cases, 42.6%). This was followed by non-conformed toxidrome with 62 cases (35.2%), which implies that the symptoms presented by the patients did not conform to any of the main toxidromes. Extrapyramidal syndrome was observed in 28 patients (15.9%). Hypoglycemic agents were used in six cases (3.4%). Anticholinergic and opiate agents were observed in two cases (1.2%), while sympathomimetic agents were less frequent, accounting for one case (0.6%).

**Figure 1:**
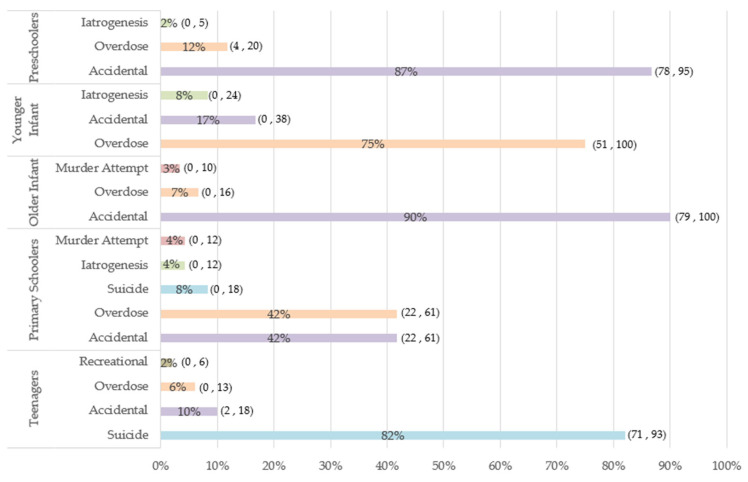
Proportions of poisoning types by age group. Note: Data are presented as proportions with confidence intervals. Information on zero-percent proportions was omitted.

Pearson’s chi-square test was performed to analyze the influence of age group on the pharmacological and toxicological variables identified in pediatric intoxications (Figure 1). A significant association was found between age group and type of intoxication (χ^2^ (20) = 185.454, *p* < 0.001). In younger infants, older infants, and preschoolers, poisoning occurs mainly as accidental events, indicating that exposure in these groups is largely unintentional. In contrast, among teenagers, the pattern changes markedly: poisonings are predominantly associated with suicide attempts, while accidental causes and overdoses occur with a lower relative frequency. Cases associated with iatrogenesis, recreational use, and homicide attempts are uncommon across all age groups and are observed only occasionally in the literature. No statistically significant associations were observed between age group and time elapsed between intoxication and medical attention (χ^2^ (24) = 25.779; *p* = 0.106) or length of stay in the emergency department (χ^2^ (8) = 7.116; *p* = 0.571).

**Figure 2:**
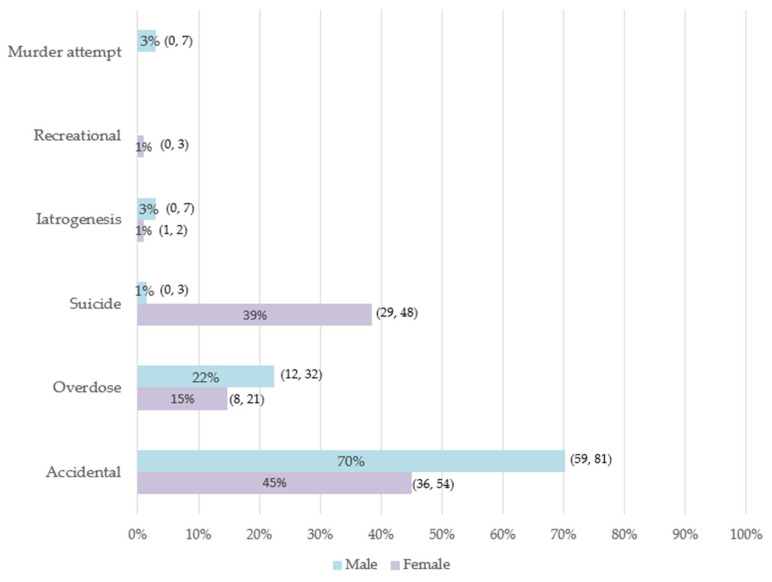
Proportions of poisoning types according to sex. Note: Data are presented as proportions and their confidence intervals. Information on zero-percent proportions was omitted.

Pearson’s chi-square test was conducted to analyze the influence of sex on the pharmacological and toxicological variables identified in pediatric intoxication cases (Figure 2). A significant association was observed between sex and type of intoxication (χ^2^ (5) = 34.439; *p* < 0.001). Accidental poisoning was the most frequent category in both sexes. However, this type of poisoning was more prevalent in men than in women. Cases related to iatrogenic, recreational, and attempted homicide intoxication were minimal in both sexes, with proportions below 5%. The findings indicate that females presented a higher proportion of suicide-related intoxications, which explains the significant differences observed between the sexes. No statistically significant associations were found between sex and the time elapsed between the onset of symptoms and the administration of medical attention (χ^2^ (1) = 0.962; *p* = 0.619) or the length of stay in the emergency department (χ^2^ (2) = 0.913; *p* = 0.633).

**Figure 3:**
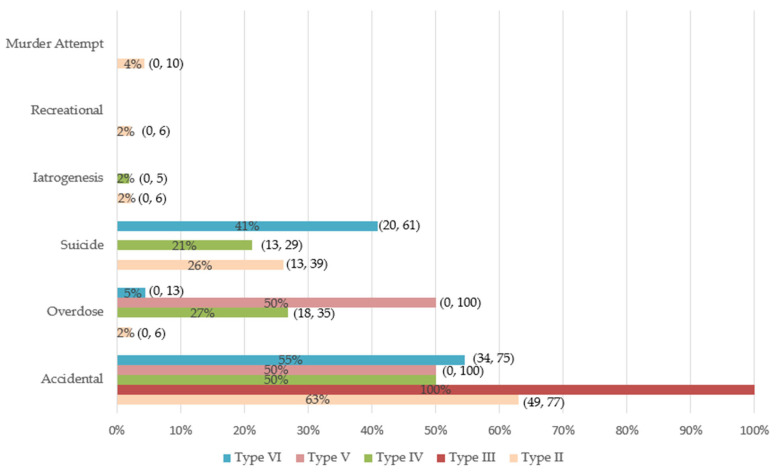
Relative percentage of drug dispensing categories and types of poisoning. Note: Data are presented as proportions and their confidence intervals. Information on zero-percent proportions was omitted from the table.

Pearson’s chi-square test was conducted to analyze the influence of the dispensing status of medications identified in intoxication cases on toxicological variables (Figure 3). A significant association was found between dispensing status and type of intoxication (χ^2^ (20) = 29.728; *p* = 0.026). Accidental intoxication predominated, accounting for half or more of the cases in dispensing status categories II, III, IV, V, and VI cases. Accidental poisoning predominated across most medication categories. This type of event is particularly notable in several categories, suggesting that the medications included are frequently involved in unintentional exposure. Overdoses were mainly concentrated in categories IV and V, whereas their presence in other categories was minimal. This indicates that certain groups of drugs are more commonly associated with consumption exceeding the recommended doses. Poisonings related to suicide attempts appeared across several medication categories, with a greater representation in category VI. This suggests that non-prescription drugs are more frequently used in these types of intentional events. No statistically significant associations were observed between dispensing status and the time elapsed between intoxication and medical attention (χ^2^ (4) = 2.842; *p* = 0.609) or length of stay in the emergency department (χ^2^ (8) = 6.365; *p* = 0.657).

### 3.2. Ordinal Logistic Regression Model

An ordinal logistic regression model was used to examine the association between age group, sex, drug classification, and length of stay in the emergency department. The model was statistically significant (χ^2^ (15) = 27.226; *p* = 0.039). The proportional odds assumption was met (Test of Parallel Lines, χ^2^ (15) = 10.373; *p* = 0.796), and the model explained 17.0% of the variance in IPED use (Nagelkerke *R*^2^ = 0.170). Multicollinearity was assessed using variance inflation factors (VIF) and collinearity diagnostics obtained from an auxiliary linear regression model that included the same predictors as the ordinal regression. The VIF values were 1.010 for the pharmacological group, with tolerance values of 0.990 for both variables. The maximum condition index was 6.845, indicating no multicollinearity. The only statistically significant predictor was the use of anticonvulsants (OR = 7.31; 95% CI: 1.993–26.762; *p* = 0.003), indicating that exposure to this drug class increased the odds of a longer stay in the emergency department by more than seven-fold compared with the reference drug. Neither age group nor sex showed significant associations (*p* > 0.05). The main parameters of the ordinal logistic regression related to the length of stay in the emergency department are presented in Table 2.

## 4. Discussion

Author medications constitute the group of toxic agents most frequently involved in acute intoxications presenting to pediatric emergency departments [23,25]. Several risk factors for acute intoxication have been identified and are commonly classified as host-related, agent-related, and environment-related factors (CENETEC).

Regarding the sex distribution of accidental intoxications, our study found a similar proportion of females and males as in previous studies. Previous studies have reported varying patterns: Puchi et al. [26] described a predominance in females, whereas Chávez Amaro [1] reported a higher frequency in males during the first few months of life. However, this proportion differed markedly in the adolescent group, where a clear predominance of females was observed, consistent with the findings of other studies [1,2,10].

When analyzing intoxication by age group, a biphasic distribution was identified, with a higher frequency in preschool-aged children and a second peak in adolescents. A previous study reported a median age of 2.1 years (IQR 1.9; *p* < 0.001) for pediatric intoxications [3], while Garrido Corro [27] also described a predominance in the preschool age group. During the preschool stage, intoxication is commonly attributed to the exploratory behavior characteristic of this developmental period [12].

During the first five years of life, children progressively increase their independence as they begin to ambulate, which, in turn, increases the risk of accidents. Factors contributing to intoxication in this age group include a tendency to imitate adult behaviors and spending more time in the home environment [1,8].

In contrast, intoxications related to suicide attempts were observed among adolescents, with a predominance of females in our study. This finding is consistent with those of previous studies [1,2,8]. The transition from childhood to adolescence represents a critical period of vulnerability, characterized by the emergence of physical, emotional, and social changes, as well as the need for autonomy and identity formation, factors that may predispose individuals to suicidal behaviors [28].

Another mechanism of intoxication identified in this study was medication overdose. During early childhood, children are completely dependent on caregivers for medication. In our study, overdoses were frequent among younger infants and school-aged children, with metoclopramide being the most commonly used drug. In a multicenter study, Garrido Corro et al. [27] reported that 65.2% of incidents were related to medication error. Similarly, ref. [29] reported that medication errors are common among patients with chronic diseases; however, this aspect was not explored in the current study. Therefore, healthcare professionals should implement educational measures to ensure correct medication dosing.

Regarding pharmacological groups, anxiolytics were the most frequently involved in intoxications, followed by the ingestion of more than one medication and the administration of antiemetics. Among anxiolytics, benzodiazepine derivatives were the most prevalent. These findings are consistent with those of Lee et al. [25] and Puchi et al. [26], who reported a predominance of hypnotics, with benzodiazepines accounting for 55% of cases and identified benzodiazepines among the leading causes. Similarly, Molina Cabrera et al. [30] described benzodiazepines as the most implicated drug group, followed by nonsteroidal anti-inflammatory drugs and antiepileptics.

Although some studies, such as that by Garrido Corro et al. [27], have reported paracetamol as the agent most frequently associated with intoxication, our findings suggest a shift in this pattern. In the present analysis, benzodiazepines predominated among psychotropic agents and appeared to surpass paracetamol as the leading cause of intoxication. Analgesics accounted for 16.1% of cases, mainly involving nonsteroidal anti-inflammatory drugs, particularly acetaminophen; this proportion was lower than that observed for benzodiazepines. Overall, intoxications involving analgesics, psychotropic drugs, cardiovascular medications, antimicrobials, and antihistamines have been widely described [3]; however, the current data highlight the growing prominence of benzodiazepines.

Several factors may have contributed to the increase in the rates of psychotropic drug poisoning among children. First, a significant increase in the prescription of psychotropic medications among the adult population has been observed in many parts of the world, especially benzodiazepines [5,31,32]. This contributes significantly to the increase in cases of poisoning from this class of drugs. Second, it is possible that because paracetamol poisoning is generally less severe than poisoning from hypnotics, some cases may have been underestimated and did not constitute medical emergencies. The study conducted by Codinach-Martín et al. [33] indicated that 85% of acetaminophen poisoning cases did not present with severe clinical signs; furthermore, doses of less than 75 mg/kg are unlikely to cause liver toxicity or produce symptoms [34].

Finally, regarding the location of exposure, 97.1% of the cases in our study occurred at home. This high prevalence of domestic poisoning events is consistent with documented practices among Mexican parents and is closely linked to regional medication storage and disposal practices. Recent evidence suggests that the Mexican population exhibits significant gaps in Knowledge, Attitudes, and Practices (KAP) regarding the disposal of expired or unused medications (EUM) [35]. Instead of utilizing specialized disposal systems, many households tend to store psychotropic drugs indefinitely, creating a persistent risk of accidental exposure in children. Furthermore, this cultural tendency toward ‘leftover storage’ is often driven by the intention of future self-medication, frequently based on recommendations from non-healthcare sources [36]. Such practices significantly increase the probability of accidental ingestion or misuse of potent drugs, such as benzodiazepines and anticonvulsants, in the domestic environment.

Garrido-Corro [27] reported that two-thirds of intoxication episodes occurred in the household, most commonly in the kitchen (27.2%). Several studies have reported that medication-related intoxications occur predominantly in the home environment, with proportions ranging from 83.7% to 94.7% [8,10,13,25,28].

The predominance of benzodiazepines as the leading causative agent in our study can be attributed to a convergence of epidemiological and social factors in the region. First, home availability is a critical driver; it is evident that these drugs are common components of the family medicine cabinet. Second, there is a duality of risk across age groups: while exposure in preschoolers is typically accidental due to exploratory behavior, benzodiazepines were the method of choice in suicide attempts among adolescents, who accounted for 24.4% of the total cases. This pattern differs from the findings in high-income countries, where acetaminophen often leads to pediatric poisoning. In Western Mexico, the combination of adult chronic prescriptions and inadequate medication transitions these drugs from a therapeutic tool to a significant pediatric hazard [37].

The time elapsed from toxic ingestion to admission to the emergency department was within the first four hours in 50% of the cases. Puchi [26] reported a median time of 60 min (IQR, 20–120 min) between the intoxication event and the first emergency consultation in their study. In contrast, ref. [10] found a mean time of 8.2 h (SD ± 13.9 h) from the event to arrival at the emergency department.

A toxidrome was identified in only 64.7% of the cases, with sedative–hypnotic toxidrome being the most frequent, followed by extrapyramidal manifestations. Similarly, ref. [10] reported sedative/hypnotic toxidromes in 48.7% of cases, followed by anticholinergic toxidromes in 31% of cases.

Prehospital management using traditional or home remedies was documented in 12.5% of cases, with milk administration being the most frequent practice. Decontamination measures performed by family members were documented in 2.8% of patients, including drinking water (1.7%), milk (0.7%), food intake (0.7%), and induction of vomiting (3.3%) [3].

The initial therapy for the management of intoxicated patients aims to reduce toxin absorption through decontamination measures and counteract the effects of the substance using antidotes. Several interventions are routinely employed, including activated charcoal administration and gastric lavage [38].

Regarding interventions to limit absorption, gastric lavage was performed in 26.6% of cases, and activated charcoal was administered in 44.3% of cases. Activated charcoal was administered within 12 h of ingestion in 96% of the cases. Cathartics were used in only 5.6% of patients. Gastrointestinal decontamination was performed in 71.8% of the cases, with gastric lavage combined with activated charcoal in 22.6% of cases [12]. Similarly, Lee [25] described management primarily based on general decontamination and symptomatic treatment, with gastric lavage performed in 6.9% of cases and activated charcoal administered in 5.4%.

Regarding the severity of intoxication, most patients had a mild clinical course (76.7%). A total of 92.1% of patients were clinically stable upon admission, whereas 7.9% were in critical condition [10]. Lee [25] described that most cases in their study were clinically mild, with only 17.2% requiring hospitalization and 3.6% being admitted to intensive care units.

Although benzodiazepines were the most frequently used agents, our multivariate analysis revealed that anticonvulsants carried a seven-fold higher risk (OR = 7.31) of prolonged hospital stays. This finding is crucial for the clinical rigor of the study, as it distinguishes between frequency and clinical impact. Although benzodiazepine poisoning is common, it is often managed relatively quickly because of the availability of specific antagonists (flumazenil) and the transient nature of hypnotic-sedative syndrome (42.6%). In contrast, anticonvulsant toxicity requires more intensive neurological monitoring and carries a higher risk of severe respiratory depression or metabolic disturbances, thereby placing a greater burden on hospital resources [39].

The survival rate in our study was 100%, consistent with previous reports indicating zero mortality associated with medication-related intoxication [3,10,12,40]. In our study, the highest frequency of medication-related intoxication occurred during summer (June–August). This finding may be related to the extended school vacation period in Mexico, which likely results in children spending more time at home and consequently increasing their exposure to medications without adequate supervision. However, other studies have reported different seasonal patterns for these species. A higher proportion of intoxications was observed between July and December [41], whereas Soave Mauricio [8] identified spring (March to May) as the season with the highest proportion of fish poisoning cases. Similarly, Lee [25] documented a peak in May and a second increase between September and October of the same year. Taken together, these findings suggest that there is no clear or consistent seasonal trend in the occurrence of medication-related intoxication in the pediatric population.

Caregivers play a fundamental role in protecting the environment surrounding children to minimize potential risk. Soave [8] reported that the mother was the primary caregiver in 61.2% of cases, whereas both parents were involved in 13.5% of cases. In our study, both parents were identified as caregivers in 57.9% of cases with a secondary level of education, and the mother was primarily responsible for household activities. Regarding caregiver age and educational level. They reported a mean caregiver/mother age of 33.7 years (SD ± 6.2) and a median of 6.4 years of schooling (SD ± 3.7) [10]. The most frequent occupation was homemaker (90.8%), with the mother being the primary caregiver in 92% of cases, and the mean number of children per household was 2.1 (SD ± 1.1).

Strategies proven effective in preventing accidental intoxication highlight the importance of improving medication storage safety in conjunction with parental education, enhanced supervision, and continuous pediatric counseling. When analyzing socioeconomic status, 79.5% of the cases of intoxication in our study occurred among children from low socioeconomic strata. In contrast, ref. [4] reported that 52.8% of the cases belonged to the middle socioeconomic class. Our institution is a public hospital where most healthcare demands are associated with limited parental education and low socioeconomic status.

The safe storage of toxic substances has been shown to reduce the risk of intoxication more effectively than parental supervision alone, representing an efficient strategy for preventing poisoning [42]. In children with greater physical ability to explore their environment, caregivers must provide increased supervision and improve their storage practices. Safe storage practices include the use of child-resistant pill containers [30] and the implementation of educational campaigns to ensure that medications are securely stored in households and kept out of children’s reach [27].

Families play a crucial role in childcare and are key factors in the prevention of poisoning, which may occur throughout childhood development and growth [34]. Therefore, addressing this public health issue should prioritize prevention through caregiver education and the implementation of legislation aimed at restricting access to such medications [26].

It is essential for pediatricians to actively educate parents and caregivers about the risks of medication-related intoxication, as most cases occur in the home setting and are frequently associated with improper storage, lack of supervision and dosing errors [41,43]. Preventive counseling should begin early, particularly for children under five years of age, including infants prior to independent ambulation, by promoting safe storage practices, restricted access to xenobiotics, and clear instructions regarding medication handling. Given that unsupervised exploratory behavior is common in this age group, anticipatory guidance plays a critical role in reducing preventable exposures.

Furthermore, intentional intoxication is a growing concern among adolescents, in whom medication overdose is often associated with emotional distress, self-harm behaviors and psychosocial vulnerability [44]. Early identification of mental health risk factors and timely emotional support are essential components of prevention strategies aimed at reducing suicide-related and poisoning. Finally, caregivers should be consistently reminded to verify correct medication dosages and administration schedules, as dosing miscalculations and therapeutic errors are frequent and preventable causes of pediatric intoxication [43,45,46].

From a clinical perspective, our findings have relevant implications for pediatric emergency care. The predominance of mild cases suggests that most patients can be safely managed with structured observation and supportive care, which may help optimize triage processes and resource allocation in busy emergency departments. Nevertheless, the identification of specific drug classes associated with greater clinical severity, particularly anticonvulsants, underscores the importance of early risk stratification and close monitoring in selected patients. These results also highlight the need for strengthened preventive strategies, including caregiver education on safe medication storage at home and targeted mental health screening among adolescents, to reduce accidental exposure and intentional ingestion. Collectively, these data support the integration of severity-based approaches into clinical protocols and public health interventions aimed at improving outcomes in pediatric drug poisoning cases.

This study had several limitations inherent to its retrospective design. We were unable to determine the exact source of the medications, whether they were current prescriptions, leftovers from previous treatments, or acquired without a prescription. Furthermore, as a single-center study conducted at a reference hospital, the observed patterns may reflect regional referral biases. Owing to these methodological constraints, it was not possible to identify specific risk factors that could inform targeted prevention strategies, which is an important area for future research.

Nevertheless, the use of standardized tools, such as the Poisoning Severity Score (PSS), provides a solid foundation for the international comparability of our clinical severity data.

## 5. Conclusions

This study demonstrates that acute drug poisoning in the pediatric population of Western Mexico predominantly affects preschoolers through accidental ingestion, regardless of sex or age. However, a concerning shift has been observed in adolescents, where intentional self-poisoning, particularly among females, has become the primary mechanism.

The high prevalence of benzodiazepines as the leading causative agent across all age groups suggests a significant public health challenge that is deeply rooted in the domestic environment. Our findings indicate that this phenomenon is likely driven by high regional prescription rates and a trend of medication dependence among adults, which increases the availability of these substances in households. Furthermore, inadequate disposal practices for expired or unused medications lead to the indefinite storage of high-risk drugs at home, creating a persistent hazard for children. These results emphasize the urgent need for targeted public health policies, including educational campaigns on safe medication storage and the implementation of effective disposal programs, to mitigate the risk of pediatric poisoning in Mexico.

Given that most events occur in the home environment, there is a critical need to focus on prevention and education directed at parents and caregivers. It is essential to establish health policies that include training healthcare personnel and developing informational campaigns on effective strategies to prevent this type of poisoning in the future. Parents should be encouraged to acquire and store all medications in child-resistant containers and keep them strictly out of children’s reach. Additionally, caregivers should be explicitly advised to avoid referring to medications as “candies,” as this practice significantly increases the risk of unintentional ingestion by young children. Clear and consistent communication regarding the potential risks of medications, coupled with safe storage practices and appropriate supervision, is essential for effective poisoning prevention strategies in the home environment.

## Figures and Tables

**Table 1:** Sociodemographic aspects and hospital care parameters (n = 176).

Variables	Frequency (%)
Gender	
Female	109 (61.9)
Male	67 (38.1)
Age group	
Younger infant	12 (6.8)
Older infant	30 (17.1)
Preschooler	60 (34.1)
Primary schoolers	24 (13.6)
Teenagers	50 (28.4)
Time between poisoning and treatment (hours)	
<1	17 (9.7)
1–4	71 (40.3)
4–8	34 (19.3)
8–12	13 (7.4)
12–24	21 (11.9)
>24	8 (4.6)
No information	12 (6.8)
Time spent in the emergency room (hours)	
<12	97 (55.1)
12–24	61 (34.7)
24–48	18 (10.2)
Hospital treatment	
Gastric lavage	47 (26.7)
Activated carbon	78 (44.3)
Antidote	56 (31.8)
Magnesium Sulfate	11 (6.3)

Note: Hospital treatment may exceed 100% in cases where multiple approaches are used.

**Table 2:** Logistic regression model for the effect of predictor variables on time spent in the emergency department.

Predictor Variables		B (SE)	*p*	Odds Ratio	95% CI for OR	
					Lower Limit	Upper Limit
Age group	Younger infant	−0.07 (0.83)	0.93	0.93	0.19	4.53
	Older infant	0.58 (0.49)	0.24	0.24	0.68	4.64
	Preschoolers	0.30 (0.43)	0.49	0.49	0.58	3.18
	Primary schoolers	0.86 (0.56)	0.12	0.12	0.79	7.04
	Teenagers	0 ^a^	.		.	.
Gender	Female	−0.05 (0.35)	0.89	0.95	0.48	1.88
	Male	0 ^a^	.		.	.
Pharmacological group	Analgesics	0.06 (0.69)	0.93	1.06	0.28	4.10
	Anxiolytics	0.36 (0.50)	0.46	1.43	0.55	3.74
	Antipsychotics	1.08 (0.74)	0.14	2.95	0.70	12.42
	Antiallergics	−0.46 (0.93)	0.63	0.63	0.10	4.09
	Anticonvulsants	1.99 (0.65)	<0.01	7.31	1.99	26.76
	Antiemetics	−0.64 (0.63)	0.32	0.53	0.15	1.83
	Antihypertensives	−1.37 (1.17)	0.24	0.26	0.03	2.54
	Anti-inflammatories	−1.18 (1.18)	0.28	0.31	0.04	2.58
	Antitussives and mucolytics	−0.42 (1.28)	0.75	0.66	0.05	8.41
	Hypoglycemic agents	−0.26 (0.85)	0.77	0.78	0.14	4.23
	Other	−0.32 (0.60)	0.58	0.73	0.23	2.28
	Polypharmacy	0 ^a^	.	.	.	.

Parameter estimates: Link function: Logit. ^a^: Parameter was set to zero because it was set as a reference. B = regression coefficient; SE = standard error; *p* = *p*-value; OR = odds ratio; CI = confidence interval.

## Data Availability

The original contributions presented in this study are included in the article. Inquiries should be directed to the corresponding author.
